# Supplementary figures and images for: Fermented Wheat Germ Protein with Histone Deacetylase Inhibitor AR42 Demonstrates Enhanced Cytotoxicity against Lymphoma Cells In Vitro and In Vivo
Source: Int J Mol Sci. 2024 Jul 18;25(14):7866. doi: 10.3390/ijms25147866 (PMC11277024; doi:10.3390/ijms25147866)

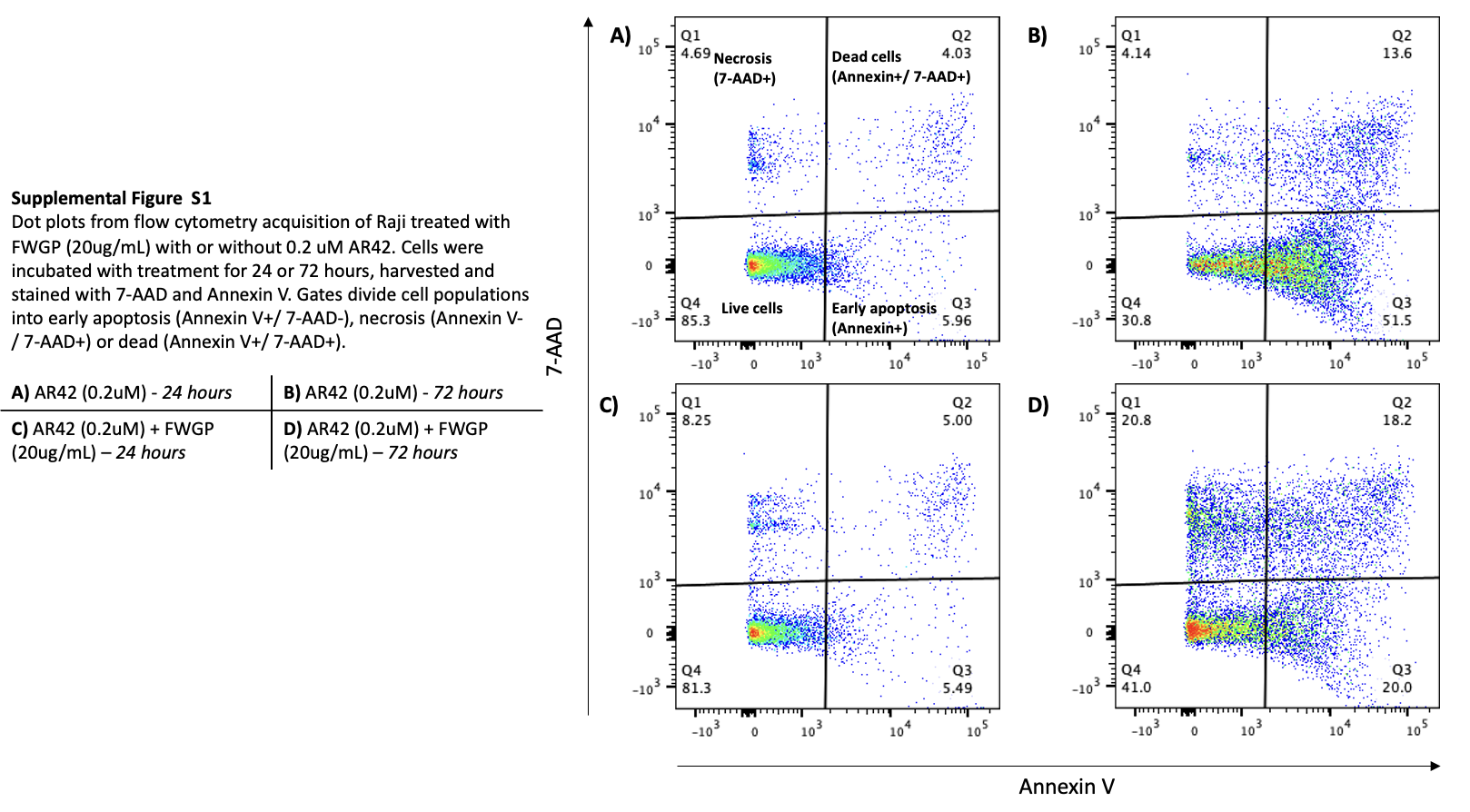

Supplement: Supplementary file 1 [file ijms-25-07866-s001.zip › Figure S1.tiff]

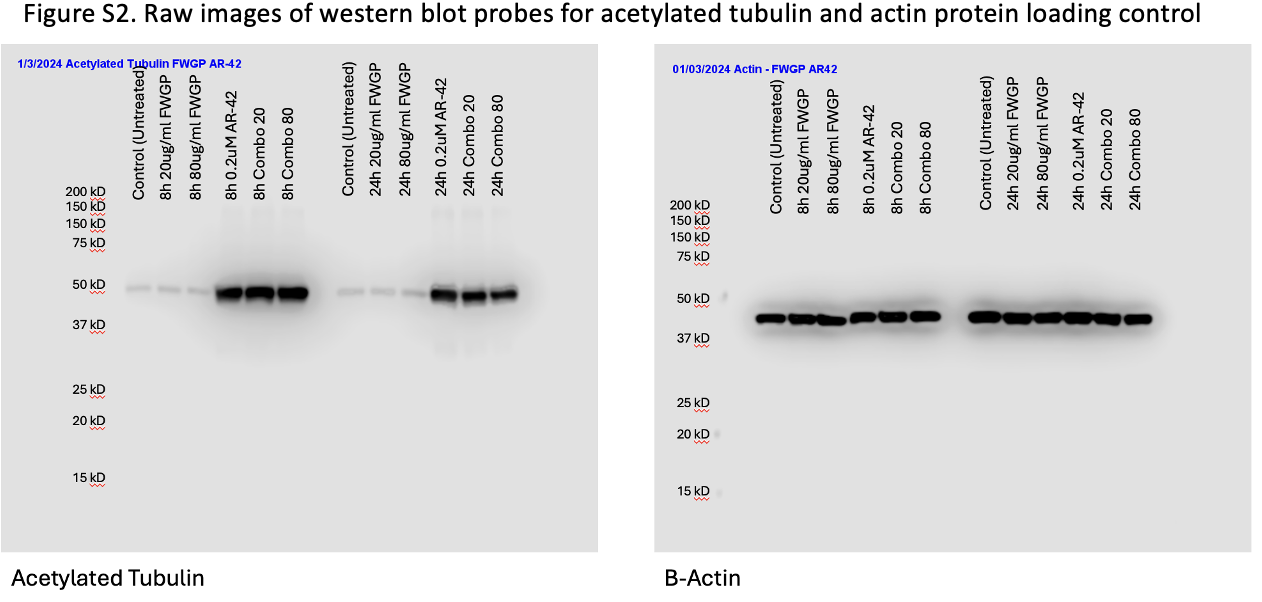

Supplement: Supplementary file 1 [file ijms-25-07866-s001.zip › Figure S2.tiff]

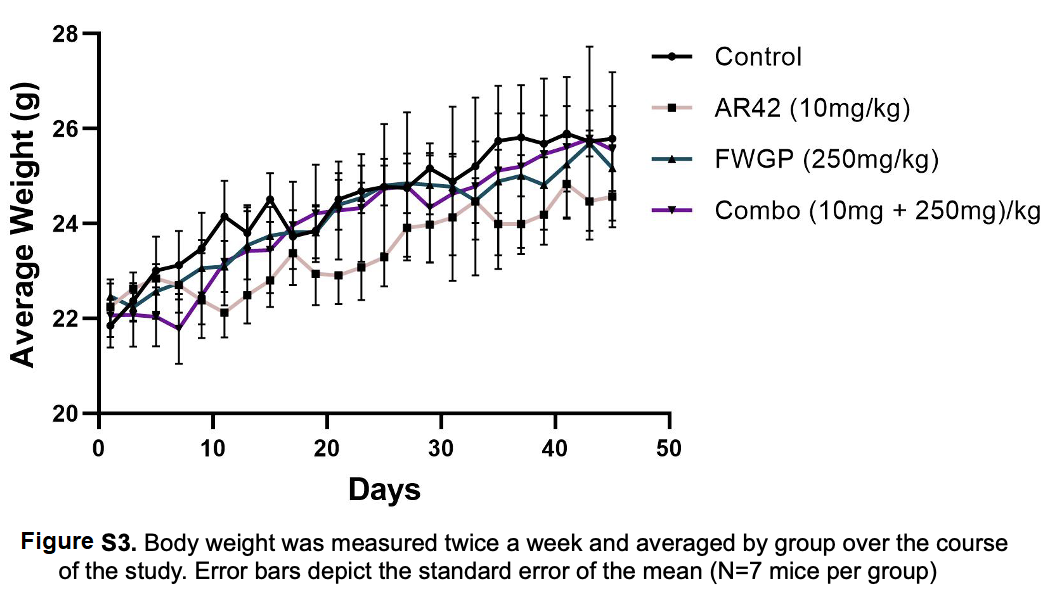

Supplement: Supplementary file 1 [file ijms-25-07866-s001.zip › Figure S3.tiff]

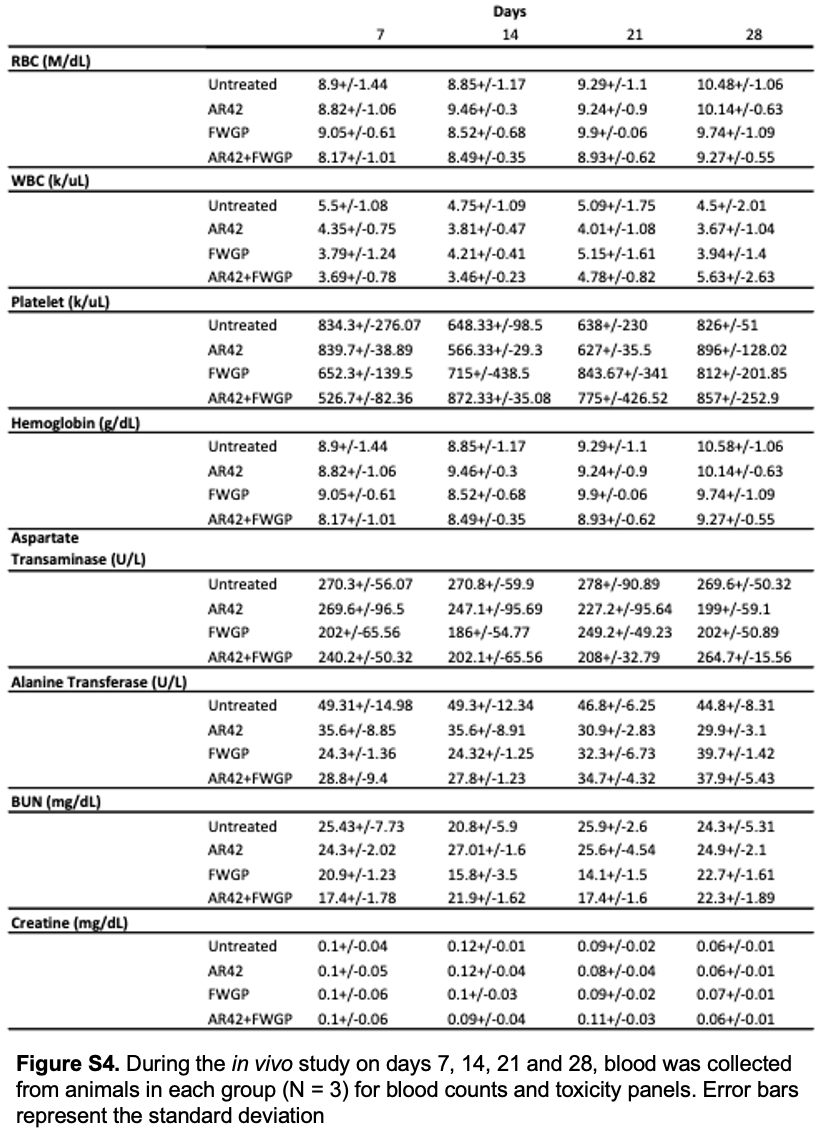

Supplement: Supplementary file 1 [file ijms-25-07866-s001.zip › Figure S4.tiff]

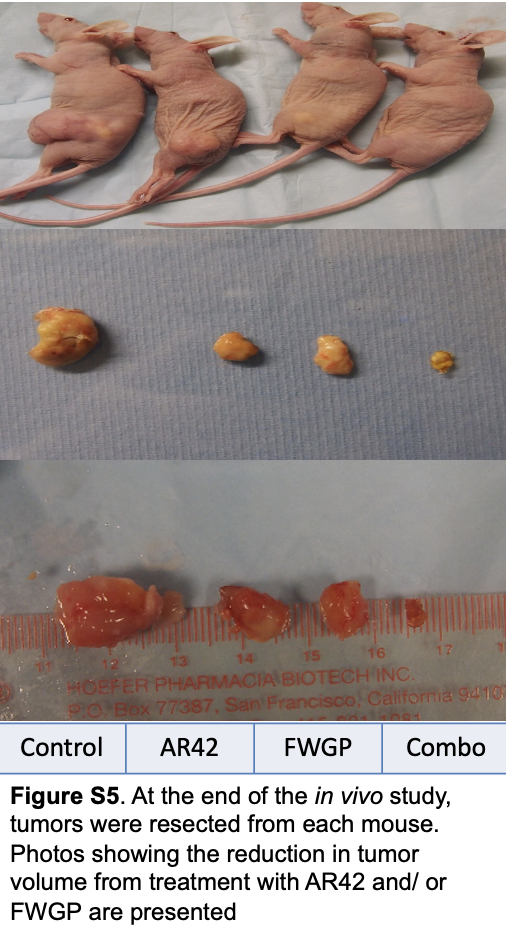

Supplement: Supplementary file 1 [file ijms-25-07866-s001.zip › Figure S5.tiff]
